# Use of Clozapine in Older Asian Patients with Schizophrenia between 2001 and 2009

**DOI:** 10.1371/journal.pone.0066154

**Published:** 2013-06-10

**Authors:** Yu-Tao Xiang, Robert W. Buchanan, Gabor S. Ungvari, Helen F. K. Chiu, Kelly Y. C. Lai, You-Hong Li, Tian-Mei Si, Chuan-Yue Wang, Edwin H. M. Lee, Yan-Ling He, Shu-Yu Yang, Mian-Yoon Chong, Ee-Heok Kua, Senta Fujii, Kang Sim, Michael K. H. Yong, Jitendra K. Trivedi, Eun-Kee Chung, Pichet Udomratn, Kok-Yoon Chee, Norman Sartorius, Chay-Hoon Tan, Naotaka Shinfuku

**Affiliations:** 1 Department of Psychiatry, Chinese University of Hong Kong, Hong Kong, China; 2 Beijing Anding Hospital, Capital Medical University, Beijing, China; 3 Maryland Psychiatric Research Center, Department of Psychiatry, University of Maryland School of Medicine, Baltimore, Maryland, United States of America; 4 School of Psychiatry and Clinical Neurosciences, University of Western Australia, Perth, Australia; 5 The University of Notre Dame Australia/Marian Centre, Perth, Australia; 6 The Key Laboratory of Mental Health, Ministry of Mental Health & Peking University Institute of Mental Health, Beijing, China; 7 Department of Psychiatry, University of Hong Kong, Hong Kong, China; 8 Department of Psychiatric Epidemiology, Shanghai Mental Health Center, Shanghai, China; 9 Department of Pharmacy, Taipei City Hospital, Taipei, Taiwan; 10 Kaohsiung Chang Gung Memorial Hospital and Chang Gung University School of Medicine, Kaohsiung, Taiwan; 11 Department of Psychological Medicine, National University of Singapore, Singapore, Singapore; 12 Hyogo Institute for Traumatic Stress (HITS), Kobe, Japan,; 13 Department of General Psychiatry, Institute of Mental Health, Buangkok View, Singapore, Singapore; 14 Department of Medicine, Alexandra Hospital/Jurong Health Services, Singapore, Singapore; 15 Department of Psychiatry, C.S.M. Medical University UP, Lucknow, Uttar Pradesh, India; 16 Department of Psychiatry, National Seoul Hospital, Seoul, Korea; 17 Department of Psychiatry, Faculty of Medicine, Prince of Songkla University, Songkhla, Thailand; 18 Department of Psychiatry and Mental Health, Tunku Abdul Rahman Institute of Neuroscience, Kuala Lumpur Hospital, Kuala Lumpur, Malaysia; 19 Association for the Improvement of Mental Health Programs, Geneva, Switzerland; 20 Department of Pharmacology, National University of Singapore, Singapore, Singapore; 21 Department of Social Welfare, School of Human Sciences, Seinan Gakuin University, Fukuoka, Japan; Baylor College of Medicine, United States of America

## Abstract

**Background:**

To date there has been no large-scale international study that examined the use of clozapine in older patients with schizophrenia. This study examined the use of clozapine and its demographic and clinical correlates in older patients with schizophrenia in East Asia during the period between 2001 and 2009.

**Method:**

Information on 1,157 hospitalized patients with schizophrenia aged 50 or older in five East Asian countries and territories (China, Hong Kong, Korea, Singapore and Taiwan) was extracted from the database of the Research on Asian Psychotropic Prescription Patterns (REAP) project. Socio-demographic and clinical characteristics and prescription of psychotropic medications were recorded.

**Results:**

Clozapine was prescribed for 20.6% of the pooled sample; 19.0% in 2001, 19.4% in 2004 and 22.9% in 2009. Multiple logistic regression analysis of the whole sample revealed that patients taking clozapine had a longer duration of illness, more negative symptoms and were less likely to receive first generation antipsychotic and anticholinergic drugs, but more likely to report weight gain compared to those not receiving clozapine. Compared to those in other sites, older patients in China were more likely to receive clozapine.

**Conclusions:**

The prescription of clozapine for older Asian schizophrenia inpatients has remained at a stable level during the past decade. The appropriateness of use of clozapine in China needs to be further explored.

## Introduction

In the past two decades a number of second-generation antipsychotic medications (SGAs) have been developed. To date there is no clear evidence that SGAs are more effective than FGAs in treating schizophrenia [1] apart from clozapine which is still the most effective antipsychotic drug for treatment-resistant schizophrenia [2]. In the past decades, prescribing patterns of clozapine have been extensively reported in patients with adult schizophrenia. In addition to its efficacy and side effects, different socio-economic situations, health care policies, prescribing traditions and clinicians' and patients' attitudes towards clozapine have influenced its prescription patterns across countries [3]. For example, clozapine was not available in Japan until the end of 2009, while in China it was one of the most commonly used antipsychotics in the past two decades (42.2% and 34.9% of schizophrenia inpatients and outpatients, respectively) [4].

Although their life expectancy is still significantly worse than that of the general population [5], nowadays, with the general improvement of psychiatric and medical care, many patients with schizophrenia live into older adulthood. In contrast to younger adults, psychotropic drug prescription for older patients must consider age-related changes in drug absorption, metabolism, and excretion and medical comorbidity. Thus, prescription patterns of clozapine observed in younger adult patients may not reflect the situation in patients with advanced age. We could not find a large-scale international study that examined the use of clozapine in older patients with schizophrenia. In order to rationalize clozapine use, it is important to understand its prescription patterns in clinical practice.

In most East Asian countries, community-based psychiatric care is not well developed and hospital-based services still dominate psychiatric care [6]. Initiated in 1999, the Research on Asian Psychotropic Prescription Pattern (REAP) project is an ongoing pharmaco-epidemiological survey on prescription trends of psychotropic drugs in schizophrenia inpatients in Asia [7], [8]. This report is based on a secondary analysis of the data of the REAP project, which was designed to: (1) examine the prescribing pattern of clozapine in Asian schizophrenia patients aged 50 years and above during the period between 2001 and 2009; and (2) explore its demographic and clinical correlates. Considering the poorer general health status of older patients, their increased vulnerability to side effects [9], [10] and the 10–15% of schizophrenia patients who are not fully responsive to FGAs [11], we hypothesized that the proportion of older Asian schizophrenia patients receiving clozapine would be less than 15%. For the purpose of this study, we defined ‘common use of clozapine’ as a figure of more than 15% in a schizophrenia patient population.

## Methods

### Settings, study design, and subjects

The first two surveys of the REAP project were conducted in July 2001 and July 2004, while the data for the third one were collected between October 2008 and March 2009. The participating countries and territories included mainland China (China hereafter), Hong Kong, Japan, Korea, Singapore and Taiwan; centres in India, Malaysia, and Thailand joined the surveys in 2009. Hospitals were selected according to convenience in the participating countries/regions. Details of the REAP project have been described elsewhere [6]. In this study, data of patients who met the following criteria in the REAP projects were analyzed: (1) a diagnosis of ICD-10 or DSM-IV schizophrenia, (2) being 50 years or older [12], (3) receiving antipsychotic treatment, and (4) having the ability to understand the study, and being willing and able to provide written or oral consent according to the requirements of the clinical research ethics committees in the respective study sites. Patients with major medical conditions were excluded.

In the REAP surveys, eligible patients were enrolled consecutively at each site. Socio-demographic and clinical information including age, gender, ethnicity, the type and dose of antipsychotic medications, benzodiazepine (BZD) and anticholinergic (ACM) drugs, length of illness, psychopathology (positive symptoms: hallucinations, delusions and thought disorder; negative symptoms: affective flattening, alogia and avolition) in the past month, extrapyramidal symptoms (EPS; including rigidity, akinesia, tremor, akathisia and dystonia) and weight gain within the past three months were collected by a review of medical records in 2001, and by either the same method or patient interviews in both 2004 and 2009 using a form designed for the study. Tardive dyskinesia (TD) is treated separately from the other forms of EPS because of its treatment characteristics. The data were collected by the patients' attending psychiatrists or by members of the research team with the agreement of the patients' treating psychiatrists.

A total of 6,761 schizophrenia inpatients were recruited in the three surveys; 2,399 from 31 psychiatric institutions, 2,136 from 25, and 2,226 patients from 50 in the 2001, 2004, and 2009 surveys, respectively. Altogether 2,236 patients fulfilled the above criteria for this report. There were only 15 patients in India, 14 in Malaysia and 8 in Thailand; in addition, in Japan clozapine was not available during the survey period. Therefore these four sites were excluded and thus 1,157 patients were included in the analyses; 405 in 2001, 319 in 2004 and 433 in 2009. In order to compare clozapine use between the current sample and younger adult patients (i.e., those under the age of 50 years) in the REAP project, figures of clozapine prescription in younger adult patients are also presented.

The study protocol was approved by the clinical research ethics committees of Chinese University of Hong Kong, Hong Kong; Peking University Institute of Mental Health, China; Taipei City Hospital, Taiwan; Chang Gung University, Taiwan; National University of Singapore, Singapore; Institute of Mental Health, Singapore; C.S.M. Medical University UP, India; National Seoul Hospital, Korea; Prince of Songkla University, Thailand; Kuala Lumpur Hospital, Malaysia; Seinan Gakuin University, Japan. Given the anonymous nature of this observational study and the minimal risk to patients, informed consent was deemed unnecessary in some study sites in line with the requirements of the local clinical research ethics committee if only a review of case notes was used. All patients receiving the interview provided written or oral consent according to the requirements of the Clinical Research Ethics Committee in the respective study sites.

### Statistical analysis

The data were analyzed using SPSS 19.0 for Windows. Comparisons of clozapine use between the three surveys were performed with chi-square test. Multiple logistic regression analysis with the “Enter” method was used to determine the demographic and clinical variables independently influencing clozapine use. Cross sectional use of clozapine was the dependent variable, while independent variables included study sites and time, age, gender, psychopathology, length of illness, use of FGAs, BZD and ACM, EPS and TD. The level of significance was set at 0.05 (two-tailed).

## Results

**Figure 1:**
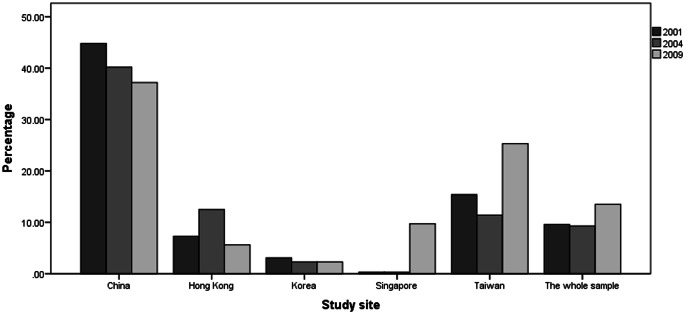
Percentage of older patients with schizophrenia receiving clozapine.

Of the 1,157 older adult patients who met study criteria, 238 (20.6%) received clozapine: 77 (19.0%) in 2001, 62 (19.4%) in 2004 and 99 (22.9%) in 2009. There was no difference between the three surveys (χ^2^ = 2.2, df = 2, p = 0.33). In contrast, 21.6% (747/3,461) of younger adult patients in the REAP project received clozapine: 20.2% (271/1,343) in 2001, 23.0% (278/1,209) in 2004 and 21.8% (198/909) in 2009. There was no significant difference in terms of prescription patterns of clozapine between the two age groups in general (χ^2^ = 0.5, df = 1, p = 0.47), or at each survey (in 2001: χ^2^ = 0.3, df = 1, p = 0.61; in 2004: : χ^2^ = 1.8, df = 1, p = 0.17; in 2009: : χ^2^ = 0.2, df = 1, p = 0.66). Figure 1 depicts the use of clozapine in older schizophrenia patients. Table 1 shows the socio-demographic and clinical characteristics and the use of clozapine in the whole sample and separately for patients by study sites.

**Table 1:** Socio-demographic and clinical characteristics of older Asian patients with schizophrenia in REAP surveys 2001–2009.

	China (n = 413)		Hong Kong (n = 75)		Korea (n = 238)		Singapore (n = 150)		Taiwan (n = 281)		Total (n = 1157)	
	N	%	N	%	N	%	N	%	N	%	N	%
Age group (yrs)												
50–59	339	82.1	48	64.0	181	76.1	99	66.0	217	77.2	884	76.4
60–69	62	15.0	20	26.7	51	21.4	46	30.7	46	16.4	225	19.4
70 and older	12	2.9	7	9.3	6	2.5	5	3.3	18	6.4	48	4.1
Length of illness (>5 yrs)	374	90.6	69	92.0	227	95.4	137	91.3	265	94.3	1072	92.7
Men	233	56.4	41	54.7	124	52.1	81	54.0	168	59.8	647	55.9
Presence of positive symptoms	209	50.6	53	70.7	164	68.9	73	48.7	190	67.6	689	59.6
Presence of negative symptoms	287	69.5	45	60.0	125	52.5	52	34.7	163	58.0	672	58.1
Presence of EPS	69	16.7	44	58.7	72	30.3	19	12.7	108	38.4	312	27.0
Presence of TD	17	4.1	14	18.7	17	7.1	6	4.0	33	11.7	87	7.5
On FGA 1	163	39.5	37	49.3	174	73.1	123	82.0	124	44.1	621	53.7
On non-clozapine SGA 2	64	15.5	17	22.7	47	19.7	6	4.0	49	17.4	183	15.8
On ACM	151	36.6	39	52.0	132	55.5	108	72.0	155	55.2	585	50.6
On BZD	54	13.1	18	24.0	100	42.0	68	45.3	71	25.3	311	26.9
Weight gain	29	7.0	11	14.7	12	5.0	0	0	25	8.9	77	6.7
On clozapine	168	40.7	6	8.0	6	2.5	3	2.0	55	19.6	238	20.6

1 any use of FGA;

2 any use of SGA; CPZeq = chlorpromazine equivalents; EPS = extrapyramidal symptoms; TD = tardive dyskinesia; FGA = first-generation antipsychotic; SGA = second-generation antipsychotic; ACM = anticholinergic medication; BZD = benzodiazepine.

Table 2 presents independent demographic and clinical correlates of clozapine prescription in older schizophrenia patients. Patients taking clozapine had longer length of illness, more negative symptoms and were less likely to receive FGAs and ACM, but more likely to report weight gain compared to those not receiving clozapine.

**Table 2:** and clinical correlates independently associated with clozapine in the combined sample (n = 1,157).

	*P* value	Odds ratio	95% C.I.
Age (year)			
50–59	–	1.0	–
60–69	0.21	0.7	0.4, 1.2
70 and older	0.06	0.4	0.1, 1.05
Length of illness (>5years)	0.002	3.6	1.6, 7.8
Male sex	0.85	1.0	0.7, 1.4
Positive symptoms	0.80	1.1	0.7, 1.6
Negative symptoms	0.04	1.5	1.03, 2.3
EPS	0.98	1.0	0.6, 1.6
TD	0.81	1.1	0.5, 2.3
On FGA	<0.001	0.2	0.1, 0.3
On BZD	0.06	0.6	0.4, 1.01
On ACM	<0.001	0.3	0.2, 0.5
Weight gain	0.008	2.4	1.3, 4.6
Study sites			
China	–	1.0	–
Hong Kong	<0.001	0.1	0.05, 0.3
Korea	<0.001	0.1	0.03, 0.2
Singapore	<0.001	0.1	0.03, 0.3
Taiwan	<0.001	0.4	0.3, 0.6
Study time			
2001 survey	–	1.0	–
2004 survey	0.16	0.7	0.4, 1.2
2009 survey	0.08	0.7	0.4, 1.05

Multiple logistic regression analysis with the non-clozapine group as the reference.

There was co-linearity between the use of FGA and non-clozapine SGAs, therefore use of non-clozapine SGA was not included in the multiple logistic regression analysis. CPZeq = chlorpromazine equivalents; EPS = extrapyramidal symptoms; FGA = first-generation antipsychotics; ACM = anticholinergic medication; BZD = benzodiazepine.

## Discussion

In this study, prescription patterns of FGAs and SGAs varied greatly across participating countries/regions (for details see Xiang et al. [13]). Our hypothesis that the proportion of older Asian schizophrenia patients receiving clozapine would be less than 15% was not confirmed. The frequency of clozapine use in the pooled sample of older patients was 20.6%, varying from 19.0% to 22.9% across surveys; the results are similar to the corresponding figures in patients under the age of 50 years in the REAP project (21.6% in the pooled sample, varying from 20.2% to 23.0% across surveys). It is noteworthy that there were great variations in clozapine use between the participating sites. The frequency of clozapine prescription was 40.7% in China and ranged between 2.5–19.6% in the other sites. The high frequency of clozapine prescription in older Chinese patients could be explained by the following reasons: (1) clozapine has continuously been used in China since its introduction in 1976 with regular blood monitoring and it has become one of the most frequently used antipsychotic drugs in China, even as the first-line treatment in some areas [14], yielding extensive clinical and research experience [3]; (2) clozapine is cheap in China costing approximately US$0.08 for 300 mg; and (3) only clozapine and risperidone were covered by basic public health insurance in most areas of China before 2005. The gradually decreasing trend of clozapine use in China (44.8% in 2001, 40.2% in 2004 and 37.2% in 2009; χ^2^ = 1.8, df = 2, p = 0.42), could be due to the introduction of strict guidelines for clozapine use and the increasing use of other SGAs. The reasons for the increased use of clozapine in 2009 in Singapore and Taiwan are unknown.

Clozapine should be used in patients unresponsive to other antipsychotic drugs [3], [15], which could explain the association of clozapine use and longer duration of illness. As negative symptoms are far more difficult to treat, they tend to occur more frequently in treatment-resistant schizophrenia. The recommended use of clozapine in this population [16] may explain the association between clozapine and negative symptoms.

In this study, adjunctive FGAs were used less frequently in the clozapine group, which is in line with current recommendations [17], [18]. There is no compelling evidence suggesting the superiority of such combinations [19]; furthermore, polypharmacy could increase the risk of adverse effects and makes it difficult to gauge which drug really works [20], [21]. People on clozapine were less likely to receive concurrent ACMs compared to non-clozapine users. This represents rational practice due to the doubtful effectiveness and elevated risk of adverse effects accompanying such co-prescription [19]. As expected, clozapine was associated with more weight gain in this study, which supports the findings of previous studies [22].

The results of this survey should be interpreted with caution due to several limitations. First, the REAP project only focuses on inpatients in selected East Asian countries and regions. The restriction of the study sample to hospitals selected according to convenience where patients were consecutively examined limits generalizability of the findings. In addition, the number of patients on clozapine was very small in some participating countries/regions, which hinders within-group comparisons between the three surveys in each site. Second, the severity of psychopathology and the drug-induced side effects were not assessed by standardized instruments. Third, the data were collected by a review of medical records in 2001 and by either a review of medical records or patient interviews in 2004 and 2009, which might have potentially led to observational bias. Fourth, some important factors likely to influence appropriate clozapine use, such as age of onset, use of prior antipsychotics, local prescription guidelines, reimbursement policies and the type of psychiatric facilities were not evaluated. There are differences in healthcare schemes, prescribing traditions and treatment guidelines between institutions even within one country, and within one institution between different study times. The confounding effects of these differences and the potential multiple interactions between them could not be explored. More advanced analyses are needed using a sophisticated design in future REAP surveys. Finally, the appropriateness of clozapine use could not be explored.

In conclusion, one fifth of older Asian schizophrenia inpatients received clozapine over the period between 2001 and 2009. Clozapine was particularly commonly prescribed in China (40.7%). Given that most treatment guidelines for schizophrenia suggests clozapine only in treatment-resistant patients [3], [16] and considering the poorer general health status of older patients and their increased vulnerability to psychotropic-induced side effects, the reasons for common use of clozapine in older patients in China warrant further investigations.
